# Humoral and cellular responses to SARS‐CoV‐2 in patients with B‐cell haematological malignancies improve with successive vaccination

**DOI:** 10.1111/bjh.18962

**Published:** 2023-07-04

**Authors:** Christopher L. Pinder, Dylan Jankovic, Thomas A. Fox, Amy Kirkwood, Louise Enfield, Aljawharah Alrubayyi, Emma Touizer, Rosemarie Ford, Rachael Pocock, Jin‐Sup Shin, Joseph Ziegler, Kirsty J. Thomson, Kirit M. Ardeshna, Dimitra Peppa, Laura E. McCoy, Emma C. Morris

**Affiliations:** ^1^ Division of Infection and Immunity University College London London UK; ^2^ Department of Clinical Haematology University College London Hospitals, NHS Foundation Trust London UK; ^3^ CR UK and UCL Cancer Trials Centre UCL Cancer Institute, UCL London UK

**Keywords:** antibodies, B cells, haematological malignancies, infection, vaccines, T cells

## Abstract

Patients with haematological malignancies are more likely to have poor responses to vaccination. Here we provide detailed analysis of the humoral and cellular responses to COVID‐19 vaccination in 69 patients with B‐cell malignancies. Measurement of anti‐spike IgG in serum demonstrated a low seroconversion rate with 27.1% and 46.8% of patients seroconverting after the first and second doses of vaccine, respectively. In vitro pseudoneutralisation assays demonstrated a poor neutralising response, with 12.5% and 29.5% of patients producing a measurable neutralising titre after the first and second doses, respectively. A third dose increased seropositivity to 54.3% and neutralisation to 51.5%, while a fourth dose further increased both seropositivity and neutralisation to 87.9%. Neutralisation titres post‐fourth dose showed a positive correlation with the size of the B‐cell population measured by flow cytometry, suggesting an improved response correlating with recovery of the B‐cell compartment after B‐cell depletion treatments. In contrast, interferon gamma ELISpot analysis showed a largely intact T‐cell response, with the percentage of patients producing a measurable response boosted by the second dose to 75.5%. This response was maintained thereafter, with only a small increase following the third and fourth doses, irrespective of the serological response at these timepoints.

## Graphical abstract

Patients with haematological malignancies often present with immune dysfunction which increases the likelihood of a poor response to vaccination. Our analysis of both the humoral and cellular immune response to repeated SARS‐CoV‐2 immunisation shows a low antibody binding and neutralising response at early timepoints. However, this improves with successive doses, correlating with recovery of the B‐cell compartment following cessation of B‐cell‐depleting therapy. In contrast, the T‐cell response is largely intact and maintained thereafter, with small boosts in magnitude following the third and fourth doses.

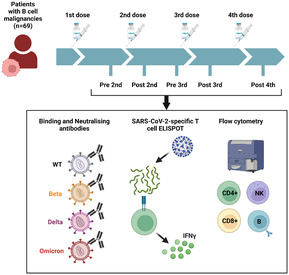

## INTRODUCTION

Outcomes for patients with haematologic malignancy infected with SARS‐CoV‐2 are poor.^1^ Although vaccination and anti‐viral therapies have dramatically reduced mortality from COVID‐19 in healthy individuals, there remain concerns that patients with haematological malignancies, particularly those who receive B‐cell‐depleting agents, may have suboptimal responses to vaccination.^2^, ^3^ In particular, these individuals are likely to have inferior vaccine responses due to both the immune dysfunction induced by the cancer and from the immunosuppressive actions of systemic anti‐cancer therapy (SACT). This places patients with B‐cell malignancies at higher risk of breakthrough infection compared to healthy individuals^4^, ^5^, ^6^, ^7^ and persistent viremia potentially associated with variant generation.^8^

Patients with B‐cell malignancies who require B‐cell‐targeted therapies to treat their disease have been shown to be particularly poor vaccine responders due to B‐cell aplasia and hypogammaglobulinaemia induced by these targeted agents. Serological responses correlate with the degree of B‐cell depletion following treatments such as rituximab.^2^, ^3^, ^9^, ^10^, ^11^ This effect is seen in terms of the magnitude of the antibody response as well as the neutralisation capacity, with patients on active treatment often showing low or absent neutralisation of wild‐type virus and novel variants.^3^, ^12^, ^13^ Therefore, it is important to understand the immune response to vaccination in this group of patients. We have previously described the serological responses to two doses of SARS‐CoV‐2 vaccine in a cohort of patients with B‐cell malignancies, primarily non‐Hodgkin lymphoma and Waldenstrom macroglobulinaemia (WM), undergoing treatment with combinations of anti‐CD20 mAbs, Bruton tyrosine kinase inhibitors (BTKi) and chimeric antigen receptor (CAR)‐T therapy,^14^ with many patients producing a spike‐binding but non‐neutralising serological response. Given the weak humoral responses observed, it was also critical to delineate all facets of the immune response after additional vaccine doses, such as immune cell phenotypes, anti‐viral T‐cell function, antibody titres, neutralisation magnitude and breadth. Many studies in this and related populations have focused on the serological response to vaccination, with fewer on the cellular component of the response. In addition, less is known about the effect of a fourth dose of vaccine on both serological and cellular responses in these patients.

Here, by combining the results of standard clinical serological measurements with in vitro neutralisation, flow cytometric phenotyping of immune cells and interferon gamma (IFNγ) ELISpot assays, we provide a detailed view of the immune response to vaccination after four doses in our previously described cohort. This enables identification of risk factors for limited seroconversion and neutralisation breadth across multiple variants of SARS‐CoV‐2 in response to vaccination.

## MATERIALS AND METHODS

### Ethics statement

This study was reviewed and received ethical approval by the South Central Berkshire B Research Ethics Committee and UK Health Research Authority approval IRAS number: 294547.

### Study recruitment

Patients receiving treatment or who had received treatment in the last 24 months for a B‐cell malignancy and receiving either the BNT162b2 (Pfizer‐BioNTech) or ChAdOx1 nCoV‐19 (Oxford‐AstraZeneca) vaccines were eligible for recruitment. 50% were receiving SACT at the time of vaccination. HIV‐positive individuals were not eligible for recruitment. Patients were recruited from both University College Hospital (UCH) and HCA UK at UCH with informed consent.

### Sample collection and processing

Blood samples were taken (where possible) prior to the second vaccination (pre‐2nd), 1 month following the second vaccination (post‐2nd), 6 months following the second vaccination (pre‐3rd), 1 month following the third vaccination (post‐3rd) and 3 months following the fourth vaccination (post‐4th). Blood was collected into both EDTA and serum clot‐activator Vacutainer tubes (BD Diagnostics). Blood samples from each timepoint were submitted for a full blood count and analysis of lymphocyte subsets (CD3, CD4, CD19, CD56) by flow cytometry (Aquios flow cytometers; Beckman Coulter). Remaining blood samples were then processed for isolation of peripheral blood mononuclear cells (PBMCs) by density gradient centrifugation and cryopreserved in 10% dimethyl sulfoxide‐containing freezing medium. Serum samples were processed and screened for anti‐SARS‐CoV‐2 antibodies against both the nucleocapsid (N) antigen and the spike (S) protein using Elecsys double antigen sandwich assays (Roche). Serum positivity was set at a threshold of ≥0.8 U/mL as per the manufacturer's instructions. Five of our cohort either had a recorded SARS‐CoV‐2 infection prior to the start of the study or had a positive result in the anti‐N screening and these individuals are shown throughout as black triangles. Other patients developing SARS‐CoV‐2 infection during the study are also indicated with black triangles at all timepoints following the recorded date of infection. The number of samples analysed for each assay and timepoint is provided where appropriate.

### In vitro pseudovirus neutralisation assay

Pseudovirus was produced as HIV‐1 particles displaying SARS‐CoV‐2 spike in HEK293T cells cultured in DMEM (Gibco) containing 10% foetal bovine serum (FBS; Labtech International) and penicillin/streptomycin (Gibco) at concentrations of 100 U/mL and 100 µg/mL, respectively (D‐10). Cells were transfected using PEI‐Max with equal amounts of HIV‐1 pCSLW luciferase reporter vector, HIV p8.91 packaging construct and SARS‐CoV‐2 spike expression vector of the desired strain (all vectors as described in Seow et al., 2020).^15^, ^16^ Viral supernatants were harvested after 48 h and filtered through a 0.45 μm filter. Serial dilutions of patient sera were prepared in D‐10 media in white 96‐well plates before incubation with pseudovirus for 1 h in a 37°C CO_2_ incubator. HeLa cells expressing ACE‐2 receptor (provided by J.E. Voss, Scripps Institute) were then added at a concentration of 1 × 10^5^ cells/mL (100 μL per well) and incubated for 48–72 h in a 37°C CO_2_ incubator. Post‐incubation, cells were lysed using the Bright‐Glo luciferase kit (Promega) before the measurement of luminescence using a Biotek Synergy H1 plate reader. Measurements were performed in duplicate and were used to calculate reciprocal inhibitory dose 50 (ID_50_) based on a standard curve produced in GraphPad Prism using serum of known neutralisation. ID_50_ values were only calculated where at least two data points exhibited >50% neutralisation. Samples presenting a non‐quantifiable but detectable neutralisation titre were designated as neutralising if they also registered a positive antibody titre on the Elecsys assay or by anti‐spike ELISA.

### Serum ELISA

ELISAs were performed as previously described^15^, ^17^ using 96‐well MaxiSorp plates coated with either SARS‐CoV‐2 spike S1 protein (provided by P. Cherepanov, Francis Crick Institute) diluted in PBS to a concentration of 3 μg/mL or goat anti‐human F(ab')_2_ (Jackson Immunoresearch) diluted 1:1000 in PBS. Plates were washed four times using PBS containing 0.05% TWEEN‐20 (PBS‐T) before blocking for 1 h with PBS containing 5% skimmed milk powder. Patient sera were diluted in PBS‐T containing 1% milk to dilutions ranging from 1:50 and 1:5000, then added to the S1‐coated wells in duplicate. Serial dilutions of an IgG standard were added to the anti‐human F(ab')_2_ wells for a standard curve. Plates were incubated for 2 h at room temperature before aspiration and washing as before. Goat anti‐human IgG conjugated to alkaline phosphatase (AP; Jackson Immunoresearch) diluted 1:1000 in PBS‐T with 1% milk was added to all wells and incubated for 1 h at room temperature, before washing and the addition of AP colorimetric substrate (Sigma Aldrich). Plates were incubated for 1 h before measurement of absorbance at 405 nm. S1‐specific IgG concentrations were calculated based on interpolation of the standard curve using a four‐parameter logistic (4PL) regression curve fitting model.

### IFN‐γ T‐cell ELISpot

ELISpot assay was performed as previously described in Alrubayyi et al.^18^ MultiScreen HTS PVDF ELISpot plates (Merck Millipore) were prepared by the addition of 30 uL of 70% ethanol for 1 min followed by washing with sterile PBS, then coated with anti‐IFN‐γ antibody (clone 1‐D1K; Mabtech) diluted to a concentration of 10 µg/mL in PBS and incubated at 4°C overnight. Plates were washed with PBS and blocked with RPMI medium (Gibco) containing 10% FBS and penicillin/streptomycin at concentrations of 100 U/mL and 100 µg/mL, respectively (R‐10) for at least 2 h. Cryopreserved PBMCs were thawed and rested for 2 h at 37°C in RPMI medium prepared as before, but containing 20% FBS (R‐20). Rested PBMCs were then resuspended in R‐10 medium and counted, then transferred to the blocked ELISpot plate at a concentration of 200 000 cells per well. Each PBMC sample was stimulated in duplicate wells with an overlapping (11 residue) 15‐mer peptide pool consisting of the entirety of the wild‐type spike (Miltenyi Biotech). Also included for each sample were negative control wells containing no stimulation, and positive control wells containing either a CMV pp65 overlapping 15‐mer peptide pool (Miltenyi Biotech) or phytohemagglutinin‐L (PHA‐L; Sigma Aldrich). Cells were incubated for 16–24 h at 37°C in a CO_2_ incubator before the plates were washed with PBS‐T. The plates were then incubated for 2 h at room temperature with biotinylated anti‐IFN‐γ (clone 7‐B6‐1; Mabtech) before washing and detection using Vectastain Elite ABC peroxidase reagent and ImmPACT AMEC Red peroxidase substrate (both Vector Labs). Detection reactions were stopped by washing with tap water then the plates dried overnight protected from light. Developed spots were detected and counted using a CTL ImmunoSpot S6 analyser. Results are reported as spot‐forming units (SFU) per 10^6^ PBMCs, calculated as the mean count minus the background (unstimulated) count. Samples were excluded if no response to spike, CMV pp65 or PHA‐L was seen. Representative wells are shown in Figure S1.

### Flow cytometry

Cryopreserved PBMCs were thawed and rested for 2 h at 37°C in R‐20 media prior to staining. Rested cells were harvested and resuspended in PBS before being transferred to a 96‐well plate for staining. PBMCs were first stained with antibodies against CXCR5 (BB515, clone RF8B2, BD Biosciences), CCR7 (APC/Cy7, clone G043H7, Biolegend) and CXCR3 (PE/Cy5, clone 1C6/CXCR3, BD Biosciences) at 37°C, followed by a second panel of antibodies at 4°C targeting CD3 (PerCP/eFluor710, clone SK7, Thermo Fisher), CD4 (PE/Dazzle594, clone RPA‐T4, Biolegend), CD8 (BV711, clone RPA‐T8, Biolegend), CD14 (BV510, clone M5E2, Biolegend), CD19 (APC, clone HIB19, Biolegend), CD25 (PE/Cy7, clone 2A3, BD Biosciences), CD38 (BV785, clone HIT2, Biolegend), CD45RA (AF700, clone HI100, Biolegend), CD56 (BV605, clone NCAM16.2, BD Biosciences), CD127 (BV650, clone A019D5, Biolegend) and PD‐1 (BV421, clone EH12.2H7, Biolegend), as well as a viability dye (Live/Dead Fixable Aqua, Thermo Fisher). Cells were fixed prior to acquisition using 2% paraformaldehyde (Biolegend). Samples were acquired on a BD Biosciences LSRFortessa calibrated using Rainbow beads (Biolegend).

### Statistical analysis

Primary statistical analysis was performed in Graphpad Prism V9. Unpaired analysis of different groups of patients was run using the non‐parametric Mann–Whitney *U* test. Correlations of two parameters were performed by non‐parametric Spearman's rho analysis.

## RESULTS

### Infrequent seroconversion and neutralisation after two vaccine doses

A total of 69 patients with B‐cell malignancies were recruited to this study,^14^ all on treatment or treated within the previous 24 months of starting vaccination with either the BNT162b2 (Pfizer‐BioNTech; *n* = 41) or ChA‐dOx1 nCoV‐19 (Oxford‐AstraZeneca; *n* = 26) vaccines (Table 1). In terms of diagnoses, two patients had B‐cell acute lymphoblastic leukaemia, 24 patients intermediate/aggressive non‐Hodgkin's lymphoma (diffuse large B‐cell lymphoma or mantle cell lymphoma), seven patients had chronic lymphocytic leukaemia, 16 patients had WM, 15 had indolent B‐cell lymphomas (follicular lymphoma [FL] or marginal zone lymphoma), and five had other B‐cell lymphomas (primary central nervous system lymphoma and primary mediastinal B‐cell lymphoma). Patients had received a variety of SACT regimens although 84% had received Rituximab, 23% had received a BTKi and 16% had received CAR‐T therapy. In all, 14 patients (20%) were on or had received one line of therapy. In total, 22 (32%) had received two prior lines or were receiving their second line of therapy. In all, 33 patients (48%) had received three prior lines or were receiving their third line of therapy. Two individuals received a heterologous immunisation with both the mRNA‐ and vector‐based products across the first and second vaccination timepoints. For the third dose of vaccine, all recorded individuals received BNT162b2 with the exception of one, who received Spikevax (Moderna). For the fourth dose, 25 individuals received BNT162b2, four received Spikevax and four were not recorded. We analysed the humoral response of our cohort using the Elecsys anti‐spike Ig assay (Roche) to measure the serum titre of spike‐specific antibody. Those with a titre >0.8 were considered seropositive and were subsequently tested for neutralisation of SARS‐CoV‐2 pseudotypes, as previously described.^15^, ^16^ Pseudotypes were produced with the spike from the wild‐type (Wuhan Hu‐1), Beta (B.1.351) and Delta (B.1.617.2) variants after the first and second vaccine doses to encompass the range of circulating variants during sample collection. For later timepoints (pre‐ and post‐third dose, post‐fourth dose), serum was also screened for neutralisation against an Omicron (BA.1/B.1.1.529.1) pseudotype, due to the global emergence of this variant during the sample collection time interval. Based on these assays, patient serum samples at each timepoint were designated as seronegative (SN), non‐neutralising or neutralising.

**TABLE 1:** Cohort demographics.

Patient characteristics	
Age (years), median (range)	60 (27–82)
Sex, *n* (%)	
Male	46 (66.7)
Female	23 (33.3)
Disease type, *n* (%)	
B‐ALL	2 (2.9)
Intermediate/aggressive NHL	24 (34.8)
CLL	7 (10.1)
WM	16 (23.2)
Indolent NHL	15 (21.7)
Other (NLPHL, PCNSL, PTLD)	5 (7.3)
Treatment status at start of vaccine course, *n* (%)	
On treatment	32 (46.4)
Completed <6 months prior	9 (13.0)
Completed >6 months prior	28 (40.6)
Treatment type, *n* (%)	
Anti‐CD20 mAb	58 (84.1)
BTKi	16 (23.2)
CAR‐T	11 (15.9)

*Note*: Cohort demographics and clinical parameters, including disease diagnoses (B‐ALL, B‐cell acute lymphoblastic leukaemia; CLL, chronic lymphocytic leukaemia; NLPHL, nodular lymphocyte predominant Hodgkin lymphoma; NHL, non‐Hodgkin lymphoma; PTLD, post‐transplant lymphoproliferative disorder; PCNSL, primary central nervous system lymphoma; WM, Waldenström's macroglobulinaemia).

**FIGURE 1:**
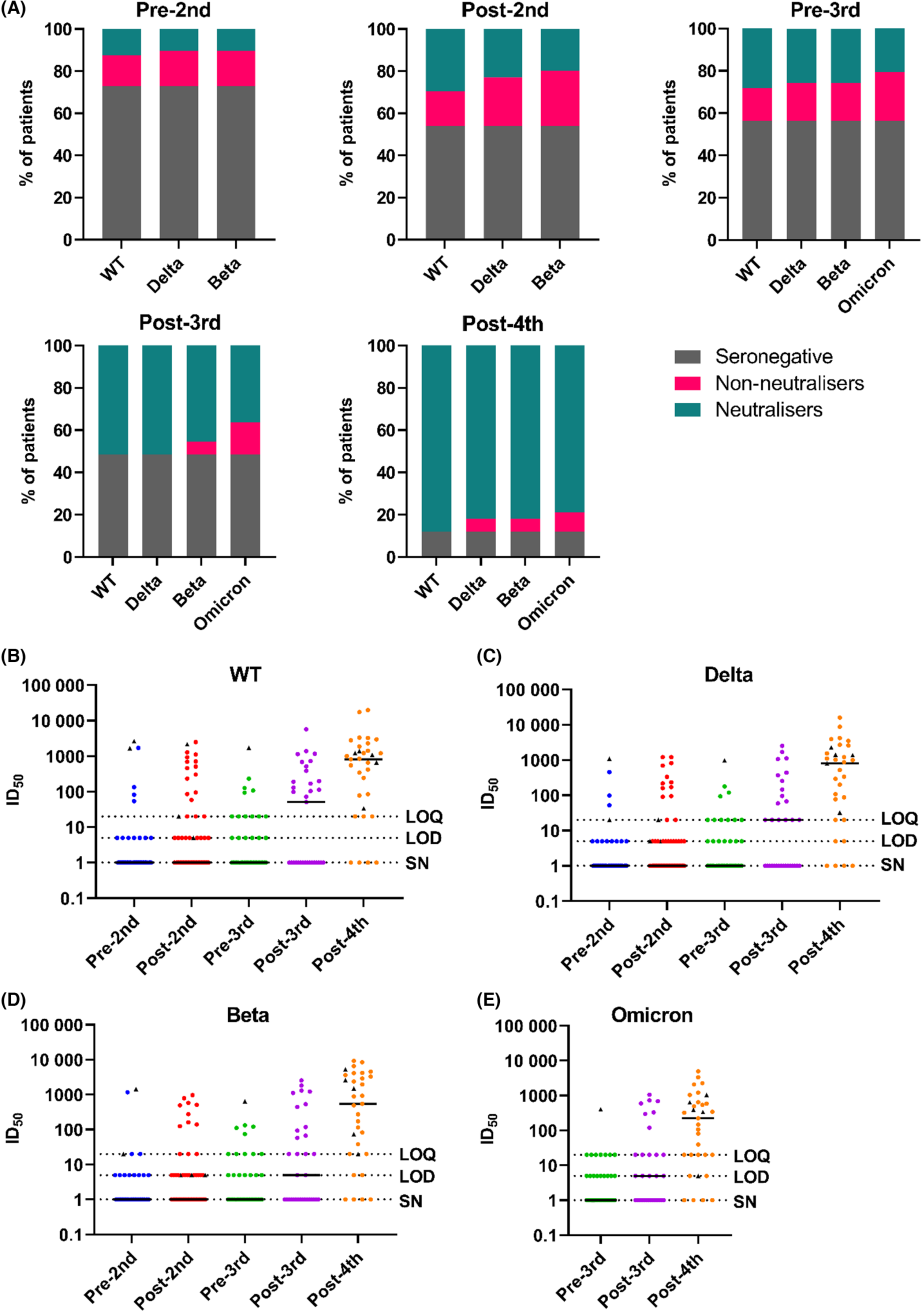
Seroconversion rates and neutralisation titres low after three vaccine doses, but largely rescued by a fourth dose. (A) Percentage of participants at the timepoints indicated above each graph, divided into seronegative, non‐neutralising and neutralising, determined for the wild‐type virus and for the variants indicated. From left to right, data are shown for pre‐second dose (*n* = 48), 1 month post‐second dose (*n* = 61), 6 months post‐second dose/pre‐third dose (*n* = 39), 1 month post‐third dose (*n* = 33) and 1 month post‐fourth dose (*n* = 33). Samples with a positive anti‐spike titre but no available sample to test for neutralisation were excluded from this analysis. (B–E) Neutralisation titres for each viral strain, measured over time. Reciprocal ID_50_ were calculated using data from duplicate serial dilutions where at least two data points exhibited >50% neutralisation. Samples which gave rise to only one data point >50% neutralisation are under the limit of quantification (LOQ) as curve‐fitting cannot be performed and were therefore assigned a value of 1:20 as this is the first dilution in the serial titration. Samples which showed neutralisation <50% at a dilution of 1:20 are below the limit of detection (LOD) and assigned a value of 1:5 to distinguish them graphically from neutralising samples below the LOQ. Seronegative (SN) samples were assigned a value of 1 for the purposes of the logarithmic scale. Patients with either a detectable anti‐N titre prior to the start of the study or with a recorded SARS‐CoV‐2 infection are indicated at all subsequent timepoints with black triangles

After a single vaccine dose, only 27.1% of patients produced spike‐specific antibodies (compared to >90% observed in healthy controls^19^, ^20^), with 12.5% also producing a detectable neutralising titre against the Wuhan strain pseudovirus (for neutralisers only, median [range] ID_50_ of 1:901 [1:54–1:2636]) (Figure 1A,B; Table S1). A second vaccine dose increased the proportion of individuals who have seroconverted to 46.7%, which is still well below the near‐complete seroconversion seen in healthy controls.^19^, ^20^ Again, most samples were unable to neutralise with only 29.5% of all patients producing a neutralising response (for neutralisers only, median [range] ID_50_ of 1:383 [1:20–1:2491]) (Figure 1A,B; Table S1). Strikingly, the proportion of samples which neutralised was even smaller when tested against SARS‐CoV‐2 variants circulating at the time of sampling. The least neutralisation was seen against the most antigenically variable variant tested at this time point, Beta, with just 19.7% producing a response (for neutralisers only, median [range] ID_50_ of 1:218 [1:20–1:971]) (Figure 1A,D; Table S1), in line with observations about this variant's relative resistance to post‐vaccination serum responses.^21^, ^22^ When measured 6 months following the second dose, the proportion of seropositive patients did not significantly decrease (47.6%) nor did the proportion of patients producing a neutralising response to the wild‐type virus (28.2%). However, neutralising titres did decrease from a median of 1:383 down to 1:20 in the ‘neutraliser’ group suggesting a substantial degree of waning over 6 months but no evidence of seroreversion (Figure 1A,B; Table S1).

### A third vaccine dose enables all seropositive individuals to produce a neutralising response

Overall, after three vaccine doses, the level of seropositivity marginally increased to 54.3% of individuals; an improved, but still inferior response compared to healthy populations.^19^, ^20^ However, the third vaccine dose boosted the neutralising response, with 51.5% of patients showing some level of neutralisation against WT (Figure 1A; Table S1). Interestingly, all seropositive individuals made a neutralising response against wild‐type virus at this time point, as was seen for the general population after only one or two vaccine doses.^23^, ^24^, ^25^ As previously observed, the more antigenically divergent variants (exemplified by Omicron, which emerged concurrently with collection of the post‐third vaccine dose samples) proved more difficult targets for neutralisation. In contrast to the improvement seen in neutralisation against WT after three vaccine doses, not all seropositive individuals could neutralise the most divergent variants (Figure 1A,D,E; Table S1). Specifically, only 45.4% and 36.4% of all samples neutralised Beta and Omicron, respectively, with relatively low potency (Figure 1D,E; Table S1), compared to that observed against wild‐type (Figure 1A; Table S1). However, this level of neutralisation was an improvement over the pre‐third dose levels, where only 25.6% and 20.5% of all patients were able to neutralise these variants, respectively (Figure 1A,D,E; Table S1).

### A fourth vaccine dose results in a neutralising response in over 88% of patients with B‐cell malignancies

Strikingly, after the fourth vaccine dose, the level of both seropositivity and the frequency of neutralising responses increased to 87.9% of the cohort (Figure 1A; Table S1), approaching the seropositivity and neutralisation seen in healthy cohorts at earlier timepoints.^23^, ^24^ As seen after the third vaccine dose, all individuals who seroconverted mounted a neutralising response against WT (Figure 1A). In addition to this increase in the proportion of individuals producing neutralising antibodies, there was a noticeable increase in the potency of neutralisation. Specifically, the median ID_50_ against WT rose from 1:198 (range 1:51–1:5740) to 1:1051 (range 1:20–1:19781) in the neutralising group, a greater than 5‐fold increase (Figure 1A,B; Table S1). However, weaker responses were again seen when considering more antigenically variable viruses, with approximately 6% of participants who had seroconverted failing to neutralise Delta or Beta (Figure 1A,C,D; Table S1). Furthermore, 10.3% of seroconverted participants failed to neutralise Omicron (Figure 1A,E; Table S1), which more accurately reflected globally circulating SARS‐CoV‐2 at the time of sampling. However, we observed a clear overall increase in the frequency of Omicron neutralisation across all participants from 36.4% after three vaccine doses to 78.8% after four vaccine doses. Moreover, there was a strong increase in average neutralisation titre between the third and fourth doses for all variants including Omicron. Infection with SARS‐CoV‐2 (depicted in Figure 1B–E with black triangles) was not sufficiently prevalent in this cohort to drive the observed increase in antibody response to the fourth dose (Figure 1B–E; Table S1). Finally, while the majority of the cohort seroconverted and made a neutralising response, there remained a subset of four patients, comprising 12.1% of all participants, who failed to seroconvert (Figure 1A; Table S1). These non‐responders did not share any common disease or treatment characteristic, comprising two patients with WM, one with CLL and one with FL.

### Functional cross‐reactive SARS‐CoV‐2 specific T‐cell responses are induced by vaccination in most patients

**FIGURE 2:**
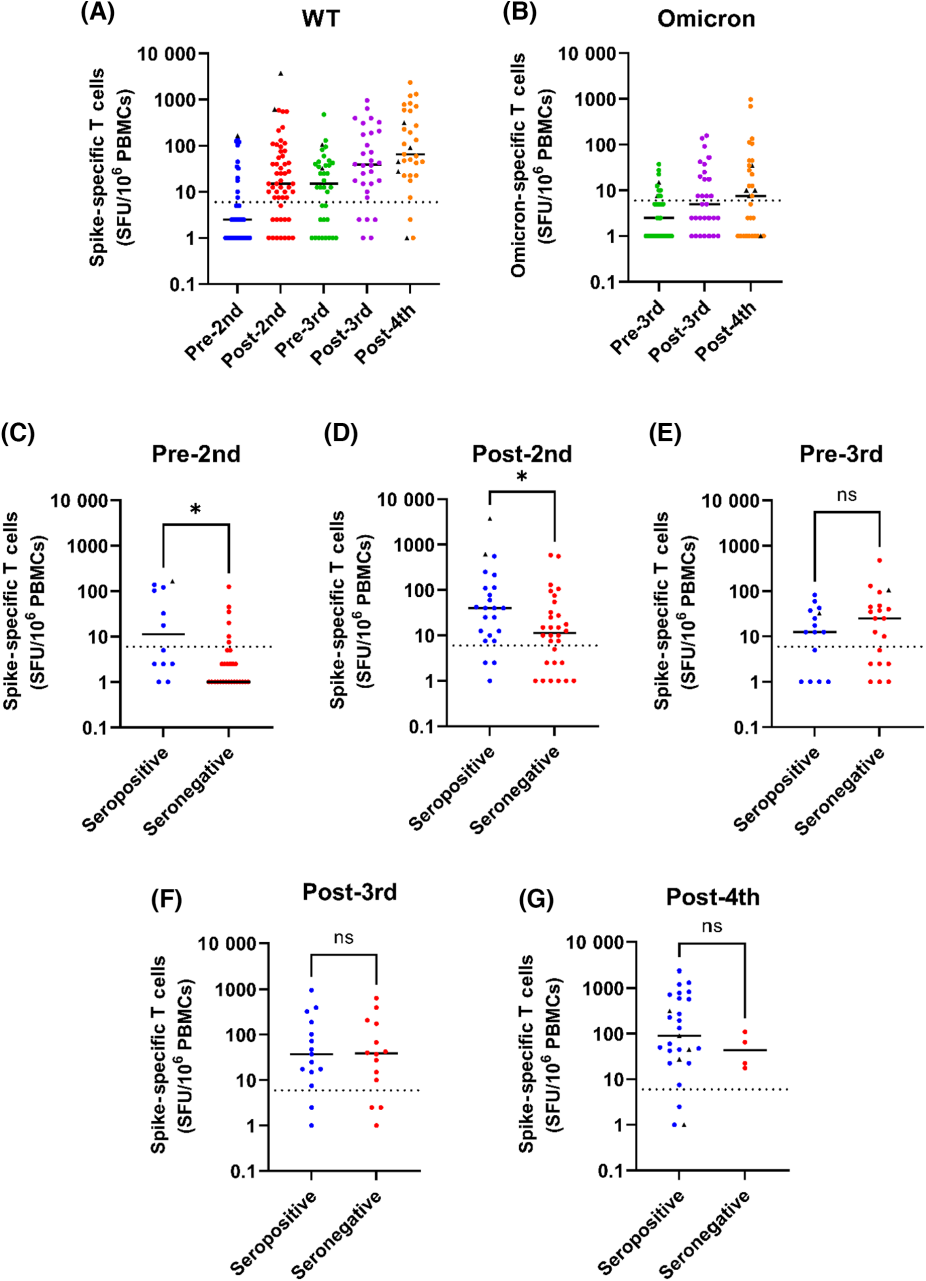
Intact T‐cell responses to vaccination, irrespective of seroconversion status. (A) Interferon gamma (IFNγ) responses to spike protein‐derived peptide pools, normalised to spot‐forming units (SFU) per 10^6^ peripheral blood mononuclear cells (PBMCs). Negative values were given a value of 1 SFU for the purposes of the logarithmic scale. ELISpot assays were run for all samples where viable PBMCs were available (*n* = 41, 53, 36, 30, 31, at each timepoint respectively). (B) IFNγ responses to the omicron mutation peptide pool pre‐ and post‐third dose, and post‐fourth dose (*n* = 35, 30, 31, respectively). (C–G) IFNγ responses to WT spike protein peptides for each timepoint, divided into seropositive and seronegative patients. ELISpot results without matched serological data were excluded from this analysis. Dashed lines indicate the threshold for a positive response, calculated as three standard deviations above the mean of all unstimulated wells. Significance is shown on each graph as calculated using the Mann–Whitney *U* test, with values of *p* = 0.0133 and *p* = 0.0297 for the Pre‐ and Post‐2nd dose timepoints respectively.

While neutralising antibodies are the canonical output of effective vaccine responses, there is emerging evidence for the role of anti‐viral T‐cell responses in control of SARS‐CoV‐2, especially in immunocompromised individuals.^26^, ^27^ Therefore, where PBMC samples were collected concurrently with clinical serum sampling, we assessed the cellular response to vaccination using an IFNγ ELISpot assay. By stimulating the PBMCs with a pool of overlapping peptides derived from the spike protein, the number of spike protein‐reactive T cells was enumerated. When examined longitudinally, spike‐specific T‐cell responses, like the antibody response, increased after the second dose of vaccine from 29.2% of patients producing a response above the threshold to 75.5% (threshold was calculated as three standard deviations above the mean of all unstimulated wells). This increase in spike‐specific T cells was maintained for up to 6th months until immediately before the third vaccine dose with no major decrease in median response, and only a slight decrease in the proportion of patients with a response above the threshold (63.9%). The third vaccine dose also increased the level of spike‐specific T cells, both in terms of median response (15 SFU to 39 SFU) and positivity (83.3%). A similar pattern was observed after the fourth dose, with an increase in the median response (65 SFU) and the positivity (90.3%) (Figure 2A; Table S2). This phenomenon of a gradual plateauing of T‐cell responses and the maintenance of the T‐cell response between the second and third doses have been previously observed in SARS‐CoV‐2‐naïve healthy controls after vaccination.^28^

We also were able to screen PBMCs against a smaller peptide pool consisting of only the regions of spike that are mutated in Omicron, to investigate whether T‐cell responses against these varying epitopes were present at the time when Omicron emerged. While Omicron‐specific T‐cell responses are smaller in magnitude compared to wild‐type full‐length spike, due to the lack of the conserved regions which have been shown to dominate T‐cell responses,^5^, ^29^ an anti‐Omicron T‐cell response can be seen prior to the third dose. A trend can be seen whereby the response to these mutated regions increases after the third dose of vaccine, increasing the percentage of patients above threshold from 28.6% to 46.7%. This further increases after a fourth dose of vaccine, with 54.8% of patients producing a T‐cell response to the Omicron‐mutated regions (Figure 2B; Table S2).

### Poor humoral response is predictive of low T‐cell response early in the vaccine response, but becomes untethered over time

Since many of these patients were able to produce a detectable spike‐specific T‐cell response but failed to seroconvert, we investigated how the two were related. Patients were divided at each timepoint into seronegative and seropositive groups, with the latter containing individuals with both neutralising and non‐neutralising activity as described above. When the T‐cell responses within these groups were examined at each timepoint, spike‐specific T cells were detected less frequently in seronegative patients prior to the second dose (Figure 2C; Table S2) with only 20.7% of seronegative patients producing a T‐cell response above the threshold, compared to 50% in the seropositive group. The magnitude was also lower with a median response below the threshold in the seronegative group compared to 11 SFU in the seropositive group. Following the second dose, more seronegative individuals had detectable spike‐specific T cells (66.7%), but the magnitude of their spike‐specific T‐cell response was again significantly lower than that of seropositive individuals (11 SFU compared to 40 SFU) (Figure 2D; Table S2), likely reflecting an overall lower magnitude immune response to vaccination than in seropositive patients. However, this association was no longer seen 6th months following the second dose where seronegative patients had detectable spike‐specific T cells at a comparable frequency to seropositive patients (65% seronegative, 66.7% seropositive), and with no significant difference in the magnitude of the response (25 SFU seropositive, 13 SFU seropositive) (Figure 2E; Table S2).

Following the third dose, there was equivalence between the seropositive and seronegative groups in terms of both the magnitude (38 SFU seropositive, 39 SFU seronegative) and frequency of T‐cell responses with 86.7% and 78.6% of patients producing a response above the threshold, respectively (Figure 2F; Table S2). After the fourth dose, the large increase in seroconversion within the cohort renders comparison of these two groups challenging. However, despite this, no significant defect is seen in T‐cell responses in the few individuals who are persistently seronegative (Figure 2G; Table S2). Together, these results suggest that a proportion of this cohort presents with a general immune dysfunction that affects the induction of both B‐ and T‐cell responses after one or two doses of vaccine. However, the T‐cell compartment can be induced to respond to the immunogen more efficiently with time and successive doses as compared to the humoral response.

### Flow cytometric analyses reveal no association between T and NK cell populations and poor serological response to vaccination

Viral‐specific components of the immune response, namely neutralising antibodies and functional T cells, have been directly associated with vaccine efficacy, but the wider immune cell profile of an individual likely underpin these specific responses. To explore this idea, we assessed the immune cell profiles using multi‐parameter flow cytometry, identifying key lymphocyte populations and multiple subpopulations of T cells, as described in the Data S1. To assess the relationship between these immune cell profiles on the humoral response to vaccination, the frequencies of each cell population were compared with the antibody response (determined by the Roche anti‐spike Ig assay, anti‐spike IgG ELISA and neutralisation titres) using a Spearman's rho correlation matrix. A threshold significance of *p* ≤ 0.01 was used to mitigate the effect of multiple comparisons.

**FIGURE 3:**
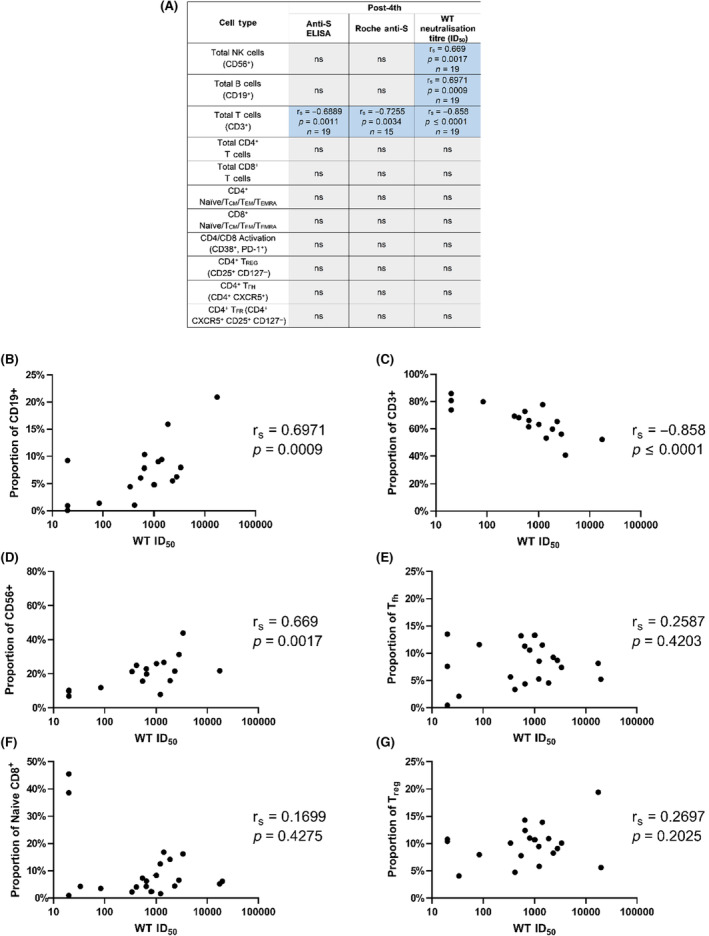
Correlations observed between serological data and flow cytometry analysis of B, NK and T‐cell subpopulations. (A) Summary table listing the correlations between each flow parameter and the Roche anti‐spike Ig, anti‐spike IgG ELISA and WT neutralisation titre at the post‐fourth dose timepoint. Significant (*p* < 0.01) correlations are listed with the Spearman's rho value (*r*_s_), the *p* value and the number of samples that were compared. Patients with CLL were excluded from comparisons of serological data with total CD3, CD19 and CD56 proportions, since expanded monoclonal CLL B cells would artificially increase the CD19 population, and thus decrease the CD3 and CD56 populations. Non‐significant correlations are listed as ‘ns’. (B–G) Representative examples of Spearman correlations, with Spearman's rho and *p* values shown. Seronegative samples are not shown on the graph due to their ‘zero’ value on the log scale for the neutralisation titres. Correlations shown are between the post‐fourth dose neutralisation titre and the flow cytometry values for proportions of (B) total B cells, (C) total T cells, (D) total NK cells, (E) T_FH_ cells as a proportion of total CD4 T cells, (F) naïve CD8 cells as a proportion of total CD8 T cells and (G) T_REG_ cells as a proportion of total non‐T_FH_ CD4 T cells.

Strikingly, the frequency of CD4^+^ and CD8^+^ T‐cell populations did not correlate with stronger or more neutralising antibody responses (Figure 3A; Table S5) across any timepoints. Nor was any association found between naïve CD8^+^ T‐cell populations and lower neutralising titres (Figure 3A,F; Table S5) as has previously been reported for other immunocompromised vaccines in subsets of this population, which are postulated to enable stronger functional T‐cell responses.^26^, ^30^ Similarly, no association was found between the frequency of circulating T_FH_ or T_REG_ cells (Figure 3A,E,G; Tables S3 and S4) and binding or neutralising antibodies. A significant, moderately sized negative association was observed between the anti‐spike IgG titres (measured by ELISA) and the proportion of T_FR_ cells at the post‐2nd dose timepoint (*r*_s_ = −0.5322, *p* = 0.0030, *n* = 29; Table S4), but this association was not observed for any other timepoints or with the Roche or neutralisation data. We did however observe several associations between serological data and larger cell populations at multiple timepoints. Interestingly, the total CD3+ T‐cell population negatively correlated with anti‐spike antibody titres (both IgG ELISA [*r*_s_ = −0.6889, *p* = 0.0011, *n* = 19] and total Ig Roche [*r*_s_ = −0.7255, *p* = 0.0034, *n* = 15]) and neutralisation titres (*r*_s_ = −0.858, *p* ≤ 0.0001, *n* = 19) following the fourth dose (Figure 3A,C; Tables S3–S5). We also observed a positive association with NK cell numbers following the fourth dose, but only with neutralisation titre (*r*_s_ = 0.669, *p* = 0.0017, *n* = 19) (Figure 3A,D).

Finally, B‐cell numbers correlated with all three serological tests at multiple timepoints, with a smaller proportion of B cells associated with a lower and more poorly neutralising antibody response. This association was observed with neutralisation titres at both the post‐3rd (*r*_s_ = 0.667, *p* = 0.0049, *n* = 17) and post‐4th (*r*_s_ = 0.6971, *p* = 0.0009, *n* = 19) timepoints (Figure 3A,B; Table S5), with anti‐spike IgG ELISA at the post‐2nd timepoint (*r*_s_ = 0.5259, *p* = 0.0058, *n* = 26) (Table S4), and with Roche titres at the post‐2nd (*r*_s_ = 0.5097, *p* = 0.0066, *n* = 27) and post‐3rd (*r*_s_ = 0.7503, *p* = 0.0035, *n* = 14) timepoints (Table S3).

Overall, these data describe a scenario where the size and quality of the antibody response is largely based on the overall health and presence of the B‐cell population, while at later timepoints where significantly fewer seronegative patients are present there is more influence on the quality of the response from other immune populations such as NK and T cells.

## DISCUSSION

In this study of 69 individuals with B‐cell malignancies, we found that seroconversion after one or two doses of SARS‐CoV‐2 vaccine was severely limited in line with previous reports.^2^, ^5^, ^14^, ^31^, ^32^, ^33^ Notably, while the spike‐specific antibody titre did substantially drop prior to the third vaccine dose, there was no evidence of seroreversion in our patients, as has been observed in some cohorts.^34^ Individuals who did seroconvert after two vaccine doses did not always produce neutralising antibody responses, unlike observations for the general population and in contrast to prior associations found between clinical anti‐spike antibody titres and neutralisation.^35^ After the third dose, this relationship was restored with all individuals who seroconverted making a neutralising response. Strikingly, seroconversion and incidence of neutralising antibodies were both increased by a fourth vaccine dose, both for wild‐type virus and the more antigenically variable Omicron variant, as has been recently suggested for a wider definition of blood cancer patients.^36^ This major improvement in the humoral response is not the result of hybrid immunity due to infection, since only a small proportion of the cohort tested positive for SARS‐CoV‐2 at any time and these individuals did not have superior antibody responses. This result demonstrates the importance of repeated boosting of these patients, especially considering a recently described correlation between poor neutralisation and breakthrough infection.^36^

The most probable determinant of poor humoral responses in these individuals is the reconstitution of their B‐cell populations following B‐cell‐depleting treatment, which routinely requires 6–9 months to begin recovery, and 9–12 months to return to normal levels.^37^ In line with this, we observed a correlation between the size of the B‐cell population and serological output (both total anti‐spike and neutralisation titres) at multiple post‐vaccine timepoints in agreement with previous findings.^12^, ^38^, ^39^ However, due to the low levels of total B cells found in these individuals, it was not feasible to explore B‐cell phenotypes which may link to weaker/absent antibody responses as performed in studies of other immunocompromised patients.^30^ Our analysis suggests that the remarkable increase in both seroconversion and antibody neutralisation after a fourth vaccine dose is driven by a higher CD19+ cell frequency rather than the number of vaccine doses. Thus, the most comprehensive vaccination plan for patients receiving B‐cell‐depleting agents may be to continue having regular boosters up until their B‐cell population reconstitutes rather than a strict adherence to a fixed number of vaccine doses. A caveat to this conclusion is that individuals undergoing B‐cell depletion in the near future may well have had prior SARS‐CoV‐2 exposure through infection or vaccination before receiving SACT which will alter the dynamics observed in serum antibody responses to SARS‐CoV‐2 vaccination.

While the humoral immune response to SARS‐CoV‐2 vaccination in this cohort is severely impaired until after the fourth vaccine dose, the anti‐spike T‐cell responses are broadly in line with that for the general population, with a marked increase in response following the second dose and a noticeable increase in magnitude with subsequent doses.^28^, ^40^, ^41^ However, the boosts have a cumulative effect with the median SFU per million PBMC gradually rising to 65 SFU after the fourth dose from 15 and 39 pre‐ and post‐third dose, respectively. The proportion of patients presenting a detectable T‐cell response also shows the same pattern, with 75.5% of patients responding after the second dose, gradually increasing to 90.3% after the fourth dose. These data largely correlate with other studies in immunocompromised groups, with more than half of patients responding after two doses followed by an increased response after a fourth dose.^3^, ^36^

Importantly, the majority of T‐cell epitopes in spike are not mutated in recent variants such as Omicron,^29^ so it is unlikely these observed responses will be limited to currently circulating variants, with data showing general equivalence between the magnitude of responses against whole wild‐type and omicron spike peptides.^5^, ^42^ However, we also observed detectable anti‐spike T‐cell responses against mutated epitopes of the omicron variant, which have been shown in other studies to be successfully induced with repeated boosting.^40^ Interestingly, the incidence of virus‐specific T‐cell responses only correlated with seroconversion after the first and second vaccine doses, suggesting that T‐ and B‐cell responses are uncoupled at later timepoints, observed in other studies of immunosuppressed patients.^3^, ^4^, ^26^ One possible explanation for this phenomenon is that while early T‐cell responses may assist in boosting antibody responses,^28^ an absence of healthy B cells may produce a certain amount of compensation of the T‐cell compartment to provide an adaptive response.^43^

Due to the heterogeneity of the cohort in terms of both the SACT regimen received and the prior lines of therapy, the number of patients receiving a particular SACT regimen were too small to correlate individual regimens with serological response. However, results were correlated with lymphocyte subset counts which correlate with SACT intensity.^44^ As previously discussed, the major association with serological output was CD19^+^ cell frequency, with correlations seen at several post‐vaccine timepoints with both the magnitude and neutralisation titre of the antibody response. We also observed a negative association between total CD3 cells and neutralising titre after the fourth dose, which may, in turn, be related to the relative change in B‐cell numbers. Similarly, we observed a positive association post‐fourth dose with neutralisation titre and NK cell frequency. NK cells can influence the development of neutralising antibodies and response to vaccination,^45^, ^46^, ^47^ and so may merit further investigation as a potential predictor of vaccine response in immunocompromised patients with repeatedly poor responses to vaccine boosting. In contrast to other studies, no correlation was observed for CD4 cell numbers and serological response,^39^ nor for naïve CD8 T cells,^26^, ^30^ the latter potentially due to the different forms of immunosuppression examined in these studies, with our cohort presenting mainly B‐cell‐focused dysfunction.

There were several limitations to our study, primarily a result of sampling limitations for our cohort. While efforts were made to acquire samples from the entire cohort at each timepoint, this was not always possible and so the data we present here should be considered cross‐sectional more than longitudinal, precluding paired analysis of many individual patients across the entire study. The demographics and clinical characteristics of our cohort may also influence our data, with one recent study showing that correlations between immune response and treatment status can differ between specific haematological malignancies.^3^ The size of our cohort also limits analysis of breakthrough infections and thus ‘protection’ levels provided by vaccination.

Despite these limitations, our data strongly demonstrate the need for repeated vaccination of B‐cell malignancy patients who are at high risk of severe COVID‐19. Our results provide evidence that repeat dosing increases the B‐cell response which we hypothesise would confer protection against severe disease and death from COVID‐19 and related complications. Our data, and those of others, strongly support repeat vaccination and targeted campaigns to encourage patients at risk to take up vaccination against COVID‐19 when offered. Our data also confirm that patients with B‐cell malignancies may remain vulnerable to severe disease and death from COVID‐19, despite vaccination. This supports the routine use of antiviral agents such as molnupiravir and remdesivir in the case of acute COVID‐19 infection in this group of patients.

## AUTHOR CONTRIBUTIONS

Christopher L. Pinder, Dylan Jankovic, Thomas A. Fox, Aljawharah Alrubayyi, Emma Touizer, Rosemarie Ford and Laura E. McCoy designed and carried out experimental work. Louise Enfield organised and acquired patient samples. Christopher L. Pinder and Amy Kirkwood performed statistical and data analysis. Thomas A. Fox, Rachael Pocock, Jin‐Sup Shin and Joseph Ziegler acquired patient data. Kirsty J. Thomson, Kirit M. Ardeshna, Dimitra Peppa, Laura E. McCoy and Emma C. Morris supervised the study. Christopher L. Pinder, Thomas A. Fox, Dimitra Peppa, Laura E. McCoy and Emma C. Morris wrote the manuscript. All authors approved the final version of the manuscript.

## FUNDING INFORMATION

This study is supported by the National Institute for Health Research (NIHR), University College London Hospitals NHS Foundation Trust (UCLH), Biomedical Research Centre and Blood Cancer UK and by UKRI MRC grant (MR/W020556/1) to LEM, DP and EM. LEM is supported by a Career Development Award (MR/R008698/1), ET by an Medical Research Council studentship (MR/N013867/1) and TF by a Wellcome Trust Clinical PhD Fellowship (216358/Z/19/Z). KMA is supported by the National Institute for Health Research (NIHR) University College London Hospitals NHS Foundation Trust (UCLH) Biomedical Research Centre.

## CONFLICT OF INTEREST STATEMENT

The authors declare no conflicts of interest.

## Supporting information


Data S1.
