# Supplementary material for: Humoral and cellular responses to SARS‐CoV‐2 in patients with B‐cell haematological malignancies improve with successive vaccination
Source: Br J Haematol. 2023 Jul 4;202(6):1091–103. doi: 10.1111/bjh.18962 (PMC10953351; doi:10.1111/bjh.18962)
Supplement: Supplementary file 1 — Data S1. [file BJH-202-1091-s001.docx]

# Supplementary Figures

Supplementary Figure 1 – Representative example of T cell ELISpot raw data

*Raw scanned well images from a representative sample measured by T cell IFNγ ELISpot. Individual wells are shown containing 200,000 PBMCs stimulated overnight with Omicron mutation-only peptides, wildtype whole-spike peptides, CMV pp65 peptides, and PHA-L. Also shown are unstimulated PBMCs, and an empty (no cell control) well.*


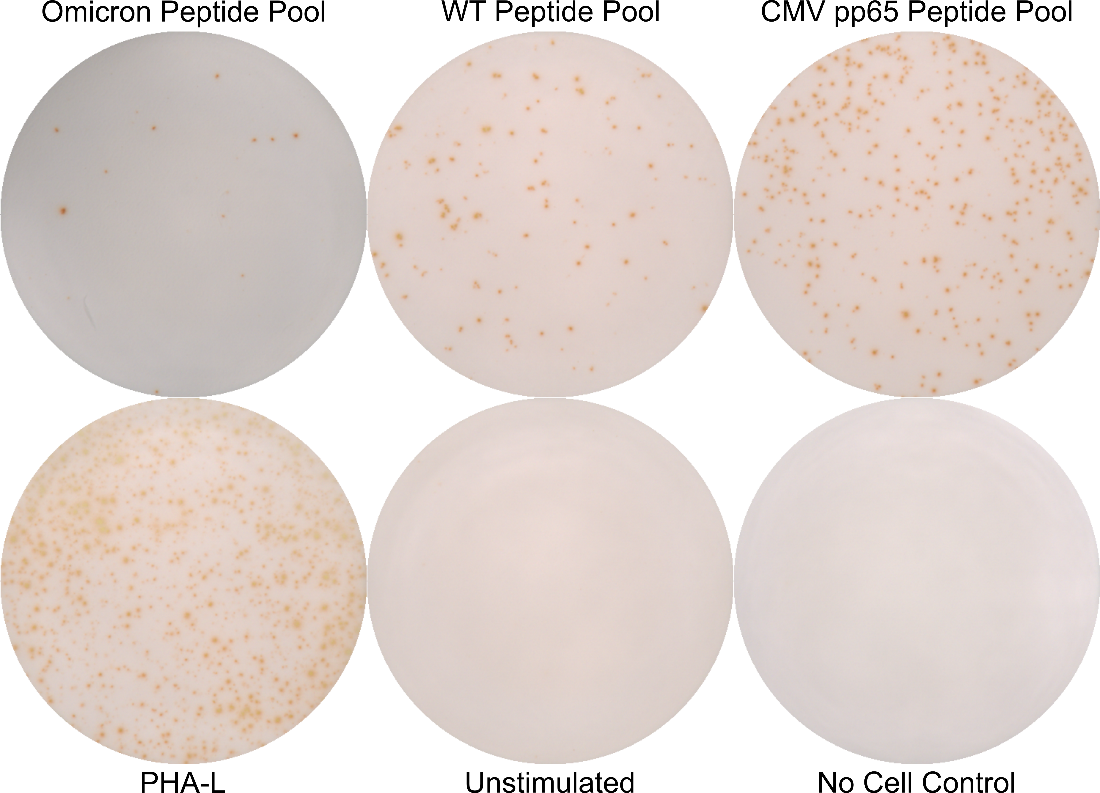


Supplementary Figure 2 – Gating strategy for identification of B cells, NK cells, and multiple subpopulations of T cells

*Detailed gating strategy for flow cytometry analysis. Total PBMCs are first gated on an FSC-A/SSC-A dot plot, followed by gating of singlets (FSC-A/FSC-H), and live lymphocytes (fixable viability dye low/negative and CD14^-^). Cells were then split into CD3^+^ (T cells) and CD3^-^ (non-T cells). Non-T cells were analysed for numbers of NK (CD56^+^) and B (CD19^+^) cells, and these numbers used to calculate the proportion of these cells as the percentage of total live lymphocytes. T cells were gated as CD4^+^ or CD8^+^. Within these two populations, cells were divided by quadrant gate into naïve (CD45RA^+^ CCR7^+^), central memory (CD45RA^-^ CCR7^+^), effector memory (CD45RA^-^ CCR7^-^) and effector memory with re-expression of CD45RA (T_EMRA_; CD45RA^+^ CCR7^-^). Both populations of T cells were also examined for expression of the activation markers PD-1 and CD38. CD4^+^ T cells were also separately gated into CXCR5^+^ and CXCR5^-^ populations, with CXCR5^+^ cells classified as circulating T follicular helper cells (T_FH_). Both this and the CXCR5^-^ population were then analysed for proportions of CD25^+^ CD127^-^ cells, classified as T follicular regulatory (T_FR_) and regulatory T cells (T_REG_) respectively. Non-T_FR_ cells were then examined for the presence of activated T_FH_ (PD-1^+^ CXCR3^-^).*


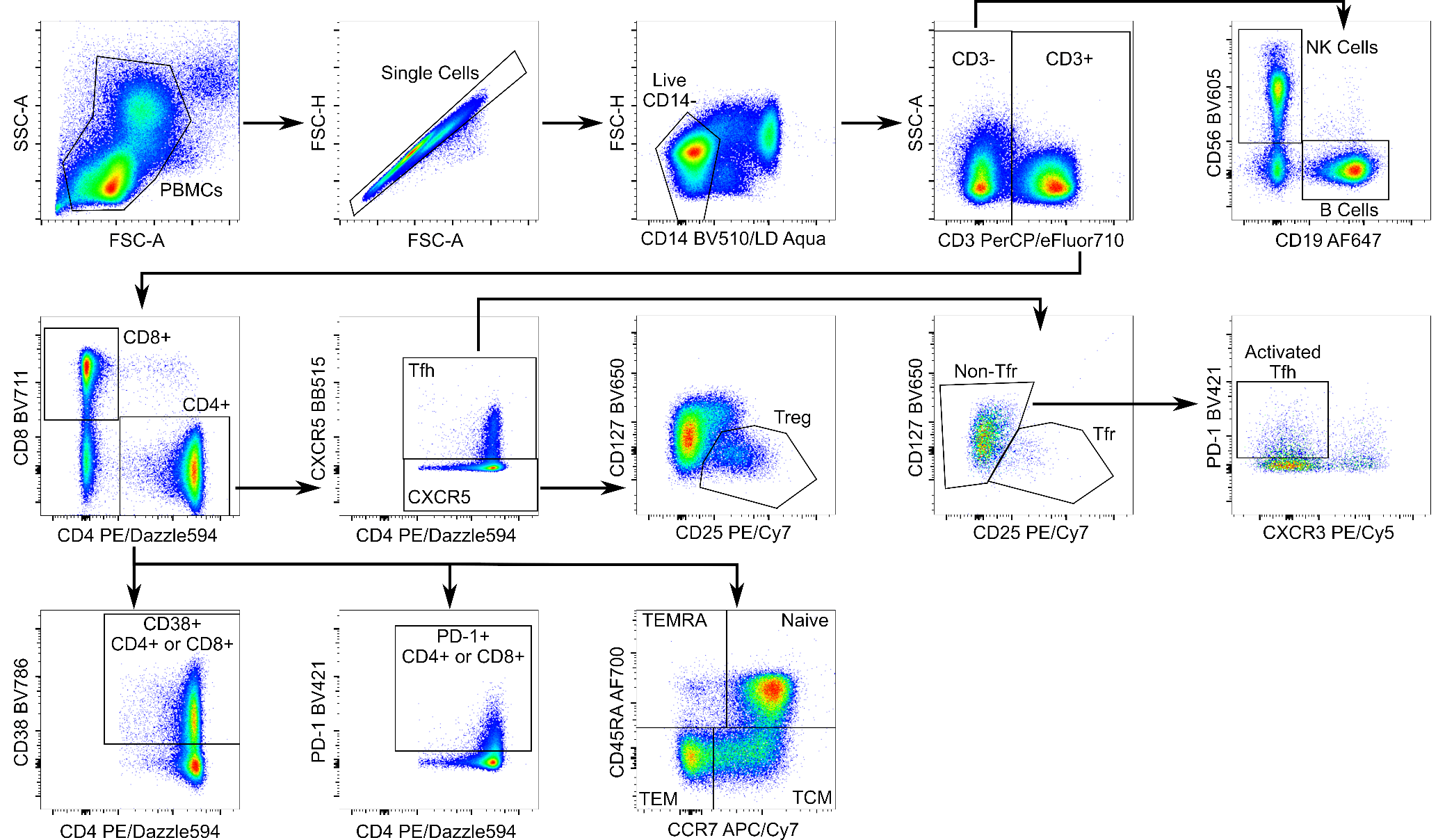


# Supplementary Tables

Supplementary Table 1 – Seroconversion and neutralisation rates, and median neutralisation titres of cohort.

*Raw data of the rates of seroconversion and neutralisation for the cohort at each timepoint, showing both the percentage (%) and numbers (n) of patients that were neutralising, seropositive but non-neutralising, and seronegative at each timepoint and for each variant of SARS-CoV-2 tested. Not included in these statistics are patients that were seropositive (determined by clinical haematology data) but for which no non-clinical serum was collected and so neutralisation status could not be determined.*

*Also shown are the median neutralisation titres (mID_50_) for each timepoint and each viral variant, as well as the number of patients (n) included in the calculation. Two sets of medians are given; one includes all seronegative and non-neutralising patients, and is the median shown by the black lines in Figure 1B-E. The other is the median IC_50_ of patients that were able to produce a measurable neutralising response, which is discussed in the text.*

| **Serostatus** | | | Pre-2nd | Post-2nd | Pre-3rd | Post-3rd | Post-4th |
| --- | --- | --- | --- | --- | --- | --- | --- |
| Neutralising | WT | % | 12.5% | 29.5% | 28.2% | 51.5% | 87.9% |
|  |  | n | 6 | 18 | 11 | 17 | 29 |
|  | Delta (B1.617.2) | % | 10.4% | 23.0% | 25.6% | 51.5% | 81.8% |
|  |  | n | 5 | 14 | 10 | 17 | 27 |
|  | Beta (B1.351) | % | 10.4% | 19.7% | 25.6% | 45.5% | 81.8% |
|  |  | n | 5 | 12 | 10 | 15 | 27 |
|  | Omicron (B1.1.529) | % |  |  | 20.5% | 36.4% | 78.8% |
|  |  | n |  |  | 8 | 12 | 26 |
| Non-Neutralising | WT | % | 14.6% | 16.4% | 15.4% | 0% | 0% |
|  |  | n | 7 | 10 | 6 | 0 | 0 |
|  | Delta (B1.617.2) | % | 16.7% | 23.0% | 17.9% | 0% | 6.1% |
|  |  | n | 8 | 14 | 7 | 0 | 2 |
|  | Beta (B1.351) | % | 16.7% | 26.2% | 17.9% | 6.1% | 6.1% |
|  |  | n | 8 | 16 | 7 | 2 | 2 |
|  | Omicron (B1.1.529) | % |  |  | 23.1% | 15.1% | 9.1% |
|  |  | n |  |  | 9 | 5 | 3 |
| Seronegative | WT | % | 72.9% | 54.1% | 56.4% | 48.5% | 12.1% |
|  |  | n | 35 | 33 | 22 | 16 | 4 |
|  | Delta (B1.617.2) | % | 72.9% | 54.1% | 56.4% | 48.5% | 12.1% |
|  |  | n | 35 | 33 | 22 | 16 | 4 |
|  | Beta (B1.351) | % | 72.9% | 54.1% | 56.4% | 48.5% | 12.1% |
|  |  | n | 35 | 33 | 22 | 16 | 4 |
|  | Omicron (B1.1.529) | % |  |  | 56.4% | 48.5% | 12.1% |
|  |  | n |  |  | 22 | 16 | 4 |
| **Median Neutralisation Response (mID_50_)** | | | | | | | |
| All Patients | WT | mIC_50_ | 1 (SN) | 1 (SN) | 1 (SN) | 1:51 | 1:818 |
|  |  | n | 48 | 61 | 40 | 33 | 33 |
|  | Delta (B1.617.2) | mIC_50_ | 1 (SN) | 1 (SN) | 1 (SN) | 1:20 | 1:800 |
|  |  | n | 48 | 61 | 40 | 33 | 33 |
|  | Beta (B1.351) | mIC_50_ | 1 (SN) | 1 (SN) | 1 (SN) | 5 (NN) | 1:545 |
|  |  | n | 48 | 61 | 40 | 33 | 33 |
|  | Omicron (B1.1.529) | mIC_50_ |  |  | 1 (SN) | 5 (NN) | 1:222 |
|  |  | n |  |  | 40 | 33 | 33 |
| Neutralisers Only | WT | mIC_50_ | 1:901 | 1:383 | 1:20 | 1:198 | 1:1051 |
|  |  | n | 6 | 18 | 11 | 17 | 32 |
|  | Delta (B1.617.2) | mIC_50_ | 1:98 | 1:195 | 1:20 | 1:96 | 1:1004 |
|  |  | n | 5 | 14 | 10 | 17 | 30 |
|  | Beta (B1.351) | mIC_50_ | 1:20 | 1:218 | 1:47 | 1:118 | 1:1214 |
|  |  | n | 5 | 12 | 10 | 15 | 30 |
|  | Omicron (B1.1.529) | mIC_50_ |  |  | 1:20 | 1:209 | 1:365 |
|  |  | n |  |  | 10 | 12 | 28 |

Supplementary Table 2 – Median T cell responses against a WT spike-derived peptide pool and an Omicron mutation-only peptide pool.

*Raw data of the median T cell responses, measured by IFNγ ELISpot, in Spot-Forming Units (SFU) per 10^6^ PBMCs. The number of patients with history of infection is also given for each timepoint. Data are shown for the whole cohort, against both peptide pools, and stratified by seropositivity. All data were calculated from ELISpots by subtraction of unstimulated wells and multiplication to a standard concentration of 10^6^ PBMCs.*

| **Total T Response (SFU/10^6^ PBMCs)** | | Pre-2nd | Post-2nd | Pre-3rd | Post-3rd | Post-4th |
| --- | --- | --- | --- | --- | --- | --- |
| WT | n | 41 | 53 | 36 | 30 | 31 |
|  | Median | 2.5 | 15 | 15 | 38.75 | 65 |
|  | Pre-Infections | 1 | 2 | 2 | 0 | 5 |
| Omicron | n |  |  | 35 | 30 | 31 |
|  | Median |  |  | 2.5 | 5 | 7.5 |
|  | Pre-Infections |  |  | 2 | 0 | 5 |
| **Stratified T Response (SFU/10^6^ PBMCs)** | | Pre-2nd | Post-2nd | Pre-3rd | Post-3rd | Post-4th |
| Seropositive | n | 12 | 23 | 15 | 15 | 27 |
|  | Median | 11.25 | 40 | 12.5 | 37.5 | 90 |
|  | Pre-Infections | 1 | 2 | 1 | 0 | 5 |
| Seronegative | n | 29 | 30 | 20 | 14 | 4 |
|  | Median | 1 (0) | 11.25 | 25 | 38.75 | 43.75 |
|  | Pre-Infections | 0 | 0 | 1 | 0 | 0 |

Supplementary Table 3 – Correlations of anti-spike Ig and lymphocyte subpopulations

*Results of a correlation matrix comparing proportions of cell subpopulations measured by flow cytometry with serological data from the Roche anti-spike Ig assay. Comparisons were made for each timepoint in the study by Spearman’s Rho with a significance cut-off of p=<0.01. Any correlations with p values above this are listed as non-significant (ns). Significant correlations are shown with the number of samples (n), the p value, and the Spearman’s Rho (r_s_) value.*

| **Cell Type** | **Pre-2nd** | **Post-2nd** | **Pre-3rd** | **Post-3rd** | **Post-4th** |
| --- | --- | --- | --- | --- | --- |
| Total CD3^+^ Cells | ns | ns | ns | ns | r_s_ = -0.7255  p = 0.0034  n = 15 |
| Total CD4^+^ T Cells | ns | ns | ns | ns | ns |
| Total CD8^+^ T Cells | ns | ns | ns | ns | ns |
| CD4^+^ Naïve (CD45RA^+^ CCR7^+^) | ns | ns | ns | ns | ns |
| CD4^+^ Central Memory (CD45RA^-^ CCR7^+^) | ns | ns | ns | ns | ns |
| CD4^+^ Effector Memory (CD45RA^-^ CCR7^‑^) | ns | ns | ns | ns | ns |
| CD4^+^ T_EMRA_ (CD45RA+ CCR7^-^) | ns | ns | ns | ns | ns |
| CD8^+^ Naïve (CD45RA^+^ CCR7^+^) | ns | ns | ns | ns | ns |
| CD8^+^ Central Memory (CD45RA^-^ CCR7^+^) | ns | ns | ns | ns | ns |
| CD8^+^ Effector Memory (CD45RA^-^ CCR7^‑^) | ns | ns | ns | ns | ns |
| CD8^+^ T_EMRA_ (CD45RA+ CCR7^-^) | ns | ns | ns | ns | ns |
| CD4^+^ T_REG_ (CD25^+^ CD127^-^) | ns | ns | ns | ns | ns |
| CD4^+^ T_FH_ (CXCR5^+^) | ns | ns | ns | ns | ns |
| CD4^+^ T_FR_ (CXCR5^+^ CD25^+^ CD127^-^) | ns | ns | ns | ns | ns |
| Activated CD4^+^ (CD38^+^ or PD-1^+^) | ns | ns | ns | ns | ns |
| Activated CD8^+^ (CD38^+^ or PD-1^+^) | ns | ns | ns | ns | ns |
| Total NK (CD56^+^) | ns | ns | ns | ns | ns |
| Total B (CD19^+^) | ns | r_s_ = 0.5097  p = 0.0066  n = 27 | ns | r_s_ = 0.7503  p = 0.0035  n = 14 | ns |

Supplementary Table 4 – Correlations of anti-spike ELISA and lymphocyte subpopulations

*Results of a correlation matrix comparing proportions of cell subpopulations measured by flow cytometry with serological data from the in-house anti-spike IgG ELISA. Comparisons were made for each timepoint in the study by Spearman’s Rho with a significance cut-off of p=<0.01. Any correlations with p values above this are listed as non-significant (ns). Significant correlations are shown with the number of samples (n), the p value, and the Spearman’s Rho (r_s_) value.*

| **Cell Type** | **Pre-2nd** | **Post-2nd** | **Pre-3rd** | **Post-3rd** | **Post-4th** |
| --- | --- | --- | --- | --- | --- |
| Total CD3^+^ Cells | ns | ns | ns | ns | r_s_ = -0.6889  p = 0.0011  n = 19 |
| Total CD4^+^ T Cells | ns | ns | ns | ns | ns |
| Total CD8^+^ T Cells | ns | ns | ns | ns | ns |
| CD4^+^ Naïve (CD45RA^+^ CCR7^+^) | ns | ns | ns | ns | ns |
| CD4^+^ Central Memory (CD45RA^-^ CCR7^+^) | ns | ns | ns | ns | ns |
| CD4^+^ Effector Memory (CD45RA^-^ CCR7^‑^) | ns | ns | ns | ns | ns |
| CD4^+^ T_EMRA_ (CD45RA+ CCR7^-^) | ns | ns | ns | ns | ns |
| CD8^+^ Naïve (CD45RA^+^ CCR7^+^) | ns | ns | ns | ns | ns |
| CD8^+^ Central Memory (CD45RA^-^ CCR7^+^) | ns | ns | ns | ns | ns |
| CD8^+^ Effector Memory (CD45RA^-^ CCR7^‑^) | ns | ns | ns | ns | ns |
| CD8^+^ T_EMRA_ (CD45RA+ CCR7^-^) | ns | ns | ns | ns | ns |
| CD4^+^ T_REG_ (CD25^+^ CD127^-^) | ns | ns | ns | ns | ns |
| CD4^+^ T_FH_ (CXCR5^+^) | ns | ns | ns | ns | ns |
| CD4^+^ T_FR_ (CXCR5^+^ CD25^+^ CD127^-^) | ns | r_s_ = -0.5322  p = 0.0003  n = 29 | ns | ns | ns |
| Activated CD4^+^ (CD38^+^ or PD-1^+^) | ns | ns | ns | ns | ns |
| Activated CD8^+^ (CD38^+^ or PD-1^+^) | ns | ns | ns | ns | ns |
| Total NK (CD56^+^) | ns | ns | ns | ns | ns |
| Total B (CD19^+^) | ns | r_s_ = 0.5259  p = 0.0058  n = 26 | ns | ns | ns |

Supplementary Table 5 – Correlations of in vitro pseudoneutralisation and lymphocyte subpopulations

*Results of a correlation matrix comparing proportions of cell subpopulations measured by flow cytometry with serological data from the in-house in vitro pseudoneutralisation assay against wildtype pseudovirus. Comparisons were made for each timepoint in the study by Spearman’s Rho with a significance cut-off of p=<0.01. Any correlations with p values above this are listed as non-significant (ns). Significant correlations are shown with the number of samples (n), the p value, and the Spearman’s Rho (r_s_) value.*

| **Cell Type** | **Pre-2nd** | **Post-2nd** | **Pre-3rd** | **Post-3rd** | **Post-4th** |
| --- | --- | --- | --- | --- | --- |
| Total CD3^+^ Cells | ns | ns | ns | ns | r_s_ = -0.8580  p = <0.0001  n = 19 |
| Total CD4^+^ T Cells | ns | ns | ns | ns | ns |
| Total CD8^+^ T Cells | ns | ns | ns | ns | ns |
| CD4^+^ Naïve (CD45RA^+^ CCR7^+^) | ns | ns | ns | ns | ns |
| CD4^+^ Central Memory (CD45RA^-^ CCR7^+^) | ns | ns | ns | ns | ns |
| CD4^+^ Effector Memory (CD45RA^-^ CCR7^‑^) | ns | ns | ns | ns | ns |
| CD4^+^ T_EMRA_ (CD45RA+ CCR7^-^) | ns | ns | ns | ns | ns |
| CD8^+^ Naïve (CD45RA^+^ CCR7^+^) | ns | ns | ns | ns | ns |
| CD8^+^ Central Memory (CD45RA^-^ CCR7^+^) | ns | ns | ns | ns | ns |
| CD8^+^ Effector Memory (CD45RA^-^ CCR7^‑^) | ns | ns | ns | ns | ns |
| CD8^+^ T_EMRA_ (CD45RA+ CCR7^-^) | ns | ns | ns | ns | ns |
| CD4^+^ T_REG_ (CD25^+^ CD127^-^) | ns | ns | ns | ns | ns |
| CD4^+^ T_FH_ (CXCR5^+^) | ns | ns | ns | ns | ns |
| CD4^+^ T_FR_ (CXCR5^+^ CD25^+^ CD127^-^) | ns | ns | ns | ns | ns |
| Activated CD4^+^ (CD38^+^ or PD-1^+^) | ns | ns | ns | ns | ns |
| Activated CD8^+^ (CD38^+^ or PD-1^+^) | ns | ns | ns | ns | ns |
| Total NK (CD56^+^) | ns | ns | ns | ns | r_s_ = 0.6690  p = 0.0017  n = 19 |
| Total B (CD19^+^) | ns | ns | ns | r_s_ = 0.667  p = 0.0049  n = 17 | r_s_ = 0.6971  p = 0.0009  n = 19 |
